# Impaired health in working children: a critical ethnography

**DOI:** 10.1186/s12889-024-19135-z

**Published:** 2024-07-15

**Authors:** Haleh Jafari, Alireza Nikbakht Nasrabadi, Nahid Dehghan Nayeri, Nematollah Fazeli, Fatemeh Khoshnavay Fomani, Shokoh Varaei

**Affiliations:** 1 Department of Medical-Surgical Nursing, School of Nursing and Midwifery, Tehran University of Medical Sciences, Tehran, Iran; 2 Institute for humanities and cultural studies, Tehran, Iran

**Keywords:** Health, Child labor, Ethnography, Qualitative research

## Abstract

Child labor is one of the important social issues that deprive children of many fundamental rights, and make them face many problems and consequences, including health problems. Thus, this study was conducted with the aim of examining the health of working children in Tehran. This is an ethnographic study that was conducted using Carspecken’s approach and was completed in 2022. The main participants of this study included working children aged 10–18 years living in Tehran. In order to collect information, the researcher was present at the workplace, school, and living places of working children for more than two years, observing their lives and activities. Formal and informal interviews were also conducted with the working children and informed people. In total, hundreds of working children were assessed and observed in this research. A friendly conversation was formed between the researcher and more than 50 children, and official interviews were conducted with six of the working children. Also, more than 10 official interviews were conducted with informed people and parents of working children. In addition to observations and interviews, documents such as medical records and drawings of working children were also examined and interpreted. The information obtained from observations, interviews, and documents was entered into MAXQDA software, and its raw codes were extracted. The high-level codes as well as sub and main categories were formed from the aggregation of low-level codes. Impaired health was formed from three subcategories of tormented body (work and environmental trauma, sexual abuse, malnutrition, fatigue, sleep disorder and inadequate hygiene), disquieted mind (anxious children, depression and isolation, reduced self-esteem and unfocused mind) and disrupted sociability (negative social role modeling, aggression and violence, stubbornness and vindictiveness, harassment and nuisance, reprehensible social behaviors, neglecting others’ ownership, disturbed relationships and out-group self-censorship). The results of the present study showed that the health of working children is compromised in various physical, psychological, and social ways. Therefore, some measures should be taken at the national and international levels to improve their health, such as revising the existing laws regarding children and informing children of their rights.

## Background

Children are not only essential assets, but also the future of a nation, and for this reason, the society must provide them with opportunities for educational, physical, psychological and social development [1]. Child labor is an important global issue that is associated with inadequate educational opportunities, poverty and gender inequality. Child labor refers to work to earn money that deprives children of their childhood, their ability and their dignity, and is harmful to their physical and mental development and health [2]. Child labor, which can be seen in various forms such as agriculture, construction work, janitor and cleaning, carpet weaving, street working, begging, and working in factories, canteens and repair shops, is a global phenomenon that is mainly seen in low-income countries [3]. Majority of working children are from Africa and Asia [4]. According to the latest global estimates, 160 million children (63 million girls and 97 million boys) were in child labor at the beginning of 2020, which is almost 1 in 10 children worldwide [4]. Some reports indicate that 61% of working children live in the Asian and Pacific regions [1]. Regarding the number of working children in Iran, there are conflicting statistics, and in unofficial reports, the number of working children in Iran has been reported between three and seven million [5]. According to Humanium, about 14% of Iranian children are forced to work in dangerous and unsanitary conditions [6].

Due to their smaller size, higher dependence and immaturity, children are more vulnerable than adults to harmful working conditions, because unhealthy environmental factors can negatively affect their growth and development [7]. Exposure to early work has important consequences on people’s physical and mental health. Working in childhood can affect people’s health in different ways. The machineries that many children work with in workshops and industry are designed for the body and mind of adults and can be dangerous for children. Working children usually need more calories while they are often faced with a lack of food resources and this can make them more vulnerable to diseases that will last a lifetime. Additionally, the lack of health and nutritional knowledge can pose many health risks to working children [8]. Work that keeps children away from home or peers, and forces them out of school can isolate these children and deprive them of social and educational opportunities. Work can also create conditions in which the child is exposed to risks, accidents, abuse and violence [9]. Child labor can have physical consequences such as bone fracture that manifests itself immediately, or have long-term affect like exposure to toxins that appears over time. It can also have various psychological consequences caused by work stress or trauma. Some studies have shown a strong connection between childhood stress and the persistence of mental disorders such as depression, anxiety, panic disorders, and schizophrenia or even health problems such as diabetes, heart disease, and immune disorders [8]. Addiction to tobacco and alcohol is high among working children, and addiction to marijuana, opium, and injectable drugs is also increasing among them [3].

Although not all types of work are harmful to the child development, Edmonds and Theoharides, citing Kassouf et al., believed that people who start work earlier often have worse health status in adulthood [8]. For example, Abdul-Ahad and colleagues, in their study of working children in Bangladesh, reported that inappropriate working conditions have caused musculoskeletal pain and skin infections in working children [10]. Ibrahim and colleagues in their study also reported that child labor is associated with a number of adverse physical and mental health outcomes, including poor growth, malnutrition, higher incidence of infectious and system-specific diseases, behavioral and emotional disorders, and reduced adaptation skills [2]. Also, many other studies have been conducted in relation to working children with different research approaches, providing valuable information to their readers. The present study is also a part of a larger research project that has been conducted with a critical ethnographic approach, to investigate the health of working children in Tehran.

## Methods

### Aim

This study, which is a part of a larger study, was conducted with the aim of examining the health of working children in Tehran.

### Design

#### Methodology

The present study is a qualitative ethnographic study that was conducted over a period of four years (more than two years in the field), using the critical ethnographic approach suggested by Carspecken [11] and was completed in 2022. The Carsepecken’s approach has three preliminary steps (creating a list of research questions, developing a list of specific items for study, and examining the researcher’s value orientations) and five main steps. The five main steps of Carspecken’s approach include.

1. Building a primary record (general observations and then purposeful observations). This step helps to produce monographic data.
2. Preliminary reconstructive analysis (analyzing monographic information using three activities of low-level coding, reconstruction of primary meaning and horizon analysis).
3. Dialogical data generation and interview.
4. Describing systemic relationships (comparing cultural content with other sites and social contexts, and discovering specific systemic relationships).
5. System relations as explanations of findings (explanation of findings based on theoretical and social models and reproduction of theory based on findings) [11].

### The study setting

The present study was conducted in different places such as work place, living place, and school of working children, according to the study objectives and the non-residential status of samples. Some of the places, where the observations and interactions took place, included Moulvi and market centers, the Working Children Support Association, Valiasr, Fatemi, Haft-e Tir, Shahrek-e Gharb, Abbas Abad and Enghelab Streets, brick kiln, flower bazaar, curtain bazaar, parks, homes of working children, and some other places. These streets are typically located in the city center, hosting numerous establishments such as markets, universities, offices, and hospitals. As a result, many working children opt to work in these bustling areas.

### Participants

The child laborers in this study predominantly hailed from neighboring countries, having migrated to Iran. Typically, their parents faced unemployment or were employed in low-income occupations, compelling the children to engage in labor due to financial constraints. Virtually all offspring within these households were part of the workforce. Female children were commonly employed on the streets until the age of 14 or 15, after which they transitioned to domestic labor. They primarily resided in densely populated, economically disadvantaged urban or peri-urban areas. Educational attainment was often lacking, with many experiencing educational delays or discontinuation.

The main participants of this study included working children (adolescents) aged 10 to 18, who lived in Tehran province and were engaged in child labor activities such as peddling, cleaning car windows, and working in workshops and shops to earn a living. If the children declined to participate in a formal or informal interview, they were excluded from this part of the study.

Another part of interactions was formed in children’s workplaces such as streets, workshops, market and flower market, etc. As a result, other people also participated in this study, the most important of whom included the families of working children, a social deputy, a manager, psychologists, social workers, physicians and healthcare officials, teachers and officials of Working Children Support Association, the person in charge of children rights convention, pedestrians, shopkeepers and welfare officials. Regarding informed people, it was necessary for these people to have at least two years of working experience with working children.

### Data collection

Before entering the field of study, a list of research question a list of specific items for study were written. In order to check the researcher value orientations, the researcher was interviewed by a member of the research team who had sufficient knowledge of the research method and research topic. In this way, the researcher’s point of view was extracted so that the research team can check and control the biases resulting from the researcher’s point of view in the analysis phase.

At the beginning of the main phase, before entering the research field, ethical approval and other necessary permissions were obtained from Tehran University of Medical Sciences and the Association for Protection of Child Laborers. During the filed study, daily observations were written and typed in a separate file immediately after leaving the research environment (working places, living places and schools of working children).

In total, hundreds of working children were assessed and observed in this research. A friendly conversation was formed between the researcher and more than 50 children, and official interviews were conducted with six of the working children (2 girls and 4 boys).

In addition, purposeful formal semi-structured interviews were also conducted with the social deputy, the school principal, two psychologists, two social workers, a physician and a health officer at the Association for the Protection of Working Children, the person in charge of Children rights convention and some of the children’s parents. Informed consent was required for all participants to participate in the study. In the case of children, in addition to the fact that the children themselves had to consent to conduct an official interview with the researcher, the consent of the parents or the school official was also obtained.

All official interviews were recorded with the interviewee’s permission and transcribed verbatim as soon as possible. Informal interviews such as friendly conversations were also coded with keywords after being transcribed at the earliest time. In addition to observations and interviews, documents such as medical records and drawings of working children were also analyzed and interpreted. To analyze the drawings of working children, we invited some working children who attended school to create drawings. Subsequently, a certified expert in drawing interpretation interpreted both the drawings and the drawing process. The prompt given to the children was to draw freely about their daily experiences or feelings. As for the interpretation, it was conducted by a certified expert in drawing interpretation, with expertise in child psychology and socio-emotional development, rather than solely a clinical psychologist. Additionally, alongside their drawings, the children were encouraged to provide verbal explanations if they wished, although this was not mandatory. To ensure the accuracy of interpretation, the expert followed established protocols for analyzing children’s drawings, incorporating both visual cues and any accompanying verbal expressions. Since the number of observations, interviews and other sources of data collection in an ethnographic study cannot be estimated in advance, data collection in this study continued until thick data was obtained. Thick data is often derived from ethnographic research, providing detailed insights into people’s lived experiences, emotions, and beliefs. After the acquisition of thick data was confirmed by experts in the field of ethnographic research methods and the issue of working children, the data collection phase was completed.

### Data analysis

The information obtained from the observations, interviews and other documents were entered into MAXQDA software and low level codes were extracted from them. For this purpose, after typing each interview and observation, by reading and carefully checking the writings, meaningful parts and low-level codes were identified and extracted. Then, the extracted codes were compared and similar codes were placed together. From the aggregation of low-level codes, high-level codes and then sub and main categories were emerged. After this stage, the findings were compared with similar social contexts and the origins of formed categories were discussed and finally, the findings were explained according to theoretical and social models.

### Validity and reliability

In this study, a comprehensive strategy was employed to ensure the validity and reliability of the data, guided by Carsepkan’s framework [11]. For this purpose, the researcher’s bias was assessed at the beginning of study and this assessment continued throughout the research. For this purpose, the researcher’s bias was assessed at the beginning of study and this assessment continued throughout the research. The process of interviewing the researcher by a knowledgeable member of the research team facilitated the scrutiny of potential biases and upheld the impartiality of the analysis phase. Initially, it provided an opportunity for the researcher to express their perspectives, values, and preconceptions openly, enabling the team to detect any potential biases or assumptions influencing data interpretation. Moreover, by asking probing questions and conducting thorough scrutiny, the interviewer could scrutinize and affirm the researcher’s perspectives, thus ensuring they were based on impartial investigation rather than personal prejudice. This meticulous approach ultimately bolstered the study’s credibility and reliability, guaranteeing that the analysis phase was carried out with utmost impartiality and thoroughness. We also established long-term relationships with participants over a span of more than two years, allowing for deep engagement and trust-building. Triangulation was employed by using multiple data collection methods to enhance the richness and reliability of our findings. Preliminary interviews were conducted with the presence of research team members to validate the interview process. Additionally, member checking was performed after each stage of analysis to mitigate any biased interpretations. Regular consultations with colleagues were undertaken to cross-validate analysis results and minimize potential biases. Furthermore, extensive interviews and informal conversations with participants and knowledgeable individuals were conducted to complement our observations. Reflective practices were woven throughout the research process, fostering transparency and reflexivity among researchers, thereby contributing to methodological refinement and the overall robustness of the study. Also, since Carsepkan emphasizes on equal power relations, in the present study, an attempt was made to prevent unequal power relations to cast a shadow on the validity of the data [11]. So, according to the Carsepkan’s recommendation, by examining and reconstructing the participants’ feedbacks, comprehensively examining the conflicts, and encouraging the participants to express self-perceptions, were tried to control and reduce unequal relations [11].

## Results

The analysis of observations in different places, the researcher’s participation in selling flowers and handkerchiefs on the street, conducting formal interviews with working children, their families and people related to them, having friendly and informal chats with working children and other people, and interpreting 10 drawings of working children helped us to collect the findings of this study. The total of 3057 low-level codes were extracted, of which about 700 codes were assigned to the main category (impaired health). The category of impaired health also consisted of three sub-categories, including tormented body, disquieted mind, and disrupted sociability. The high level codes of each sub-category are reported in Table 1.

**Table 1:** Impaired health in working children

Main category	sub-category	High level code
Impaired health	Tormented body	-Work and environmental trauma - Sexual abuse - Malnutrition - Fatigue and sleep disorder - Inadequate hygiene
	Disquiet mind	- Anxious children - Depression and isolation - Reduced self -esteem - Unfocused mind
	Disrupted sociability	- Negative social role modeling - Aggression and violence -Stubbornness and vindictiveness - Harassment and nuisance - Reprehensible social behaviors - Ignoring others’ ownership - Disrupted relationships - Out-group self -censorship

### Tormented body

The findings of this study underscore the prevalence of physical injuries among working children, reflecting the harsh realities they encounter. These injuries manifest through direct workplace hazards or environmental factors, as well as through external pressures stemming from living conditions. For instance, issues such as physical abuse, malnutrition, sleep disorders, fatigue, and inadequate hygiene significantly contribute to the tormented bodies of these children.

#### Work and environmental trauma

Work and environmental trauma emerge as significant challenges for many working children, as they often labor in non-standard environments and operate equipment designed for adults, heightening their vulnerability. Common health risks arise from this disparity, with work-related injuries and environmental hazards posing serious threats. Among the myriad injuries documented are those stemming from exposure to extreme temperatures, air pollution, and skin diseases contracted from contact with waste materials. Additionally, working children frequently endure burns, accidents, cuts, encounters with animals and vermin, as well as musculoskeletal injuries, further underscoring the precarious nature of their labor conditions.

*“M recounted the harrowing tales of his labor, each word heavy with the weight of his suffering. He spoke of a cruel man who, in a fit of rage, subjected him to a brutal assault, forcing him to scrub the car’s windows relentlessly. All this, simply because M had dared to seek recompense for his toil, by leaving a speck of dirt on the very glass he had just cleaned. It was a stark reminder of the injustices that lurked beneath the veneer of everyday life, a testament to the brutal realities faced by those who toil on the streets.”* (Informal interactions - street).

*“The initial sensation enveloping me was the searing pain in my hand, a relentless ache stemming from holding the flowers in a relatively fixed position. My weary legs echoed this agony. The cacophony of passing cars only served to exacerbate my discomfort, their ceaseless roar a relentless assault on my senses. The green light at the intersection lasts about 20 seconds. Until the light turned green, the drivers insisted on letting the others know that the light has turned green by continuously honking. Amidst the din, a throbbing headache gnawed at my temples, each pulse a symphony of pain. The acrid scent of smoke hung heavy in the air, its pungent aroma a grim testament to the urban decay that surrounded me. It felt as though a magnifying glass had been placed upon my shoes, the scorching rays of the sun concentrated upon my weary feet. Exhausted and depleted, I watched helplessly as my flowers wilted before my eyes, their vibrant hues fading into the dusk. Each wilting petal a poignant metaphor for my own dwindling strength, a silent testament to the relentless toll exacted by life on the streets.”* (observant participation- summer evening - street - Participating as a flower seller at the intersection).

#### Sexual abuse

Sexual abuse emerges as a profoundly distressing aspect affecting the health of working children, often resulting in both physical and psychological repercussions. Instances documented in our research reveal a disturbing pattern, with victims reporting ailments such as back pain and hemorrhoids following multiple assaults. In this research, we have found that these sexual abuses can be committed by the family member, stronger group members, employers and ordinary people of the society for reasons such as meeting emotional and financial needs or being defeated.

*“Children who work, not only Afghans, I never discuss nationality and race, children who work are very likely to be harmed. Even ordinary children in Iranian families often endure similar ordeals, yet it remains unspoken. For example, a year and a half ago, one of our girles was raped by two boys while at work. I was able to get a lawyer and file a complaint, but it still hasn’t worked. They arrested one of those two people, but they haven’t caught that one yet. The one who was arrested was also released on bail. I feel pain towards the judiciary. When I express my frustration to the lawyer, he brushes it off with the excuse of a heavy caseload and says, “Do you know how many cases there are?” I would say, well, it was a year and a half ago, well, that person may have done this with a few other girls, with a few other women, and now they are very easily roaming around the city. So the one who has done this and is free, what does he want to be afraid of? He says that I did this and now I am free on bail. It is not clear when this case will end and what will be their punishment? And he’s free; now he can assault me, assault others…”* (Official interview with a school principal).

*“A teenage working boy with a completely slang language said that that boys sometimes have sex with them (pointing to two teenage working girls). I said that it is not possible in the alley and the street, but he said it takes place in back alleys. I asked, do they also take money? He said, at first, they ask for money, but when we are done, we will forcefully take back the money we have given them”* (Informal interactions - street).

*“This painting shows the child’s fear and sadness. A circle is drawn around the mouth, which can resemble child abuse in the form of kissing the child, and the child is feeling angry about it”* (Interpretation of a drawing).

*“Many times the drivers show me a lot of money and say that if you come with me, I will give you this money. But well, I know what they mean and I will not go with them”* (Official interview with a working child- girl/14y).

#### Malnutrition and insufficient growth

Evidence gathered from screenings and clinical examinations highlights a distressing trend among working children, with many exhibiting stunted physical growth and suffering from malnutrition, underweight conditions, and anemia. Root causes of these health disparities are multifaceted, with factors such as family financial poverty and low literacy levels among parents and children exerting significant influence. The sheer number of children within this social group further compounds these challenges, exacerbating their vulnerability to inadequate nutrition and hindered growth. Addressing these underlying factors is paramount in mitigating the pervasive health risks faced by working children and fostering their holistic development.

*“How frequently does your family consume chicken? Once a month. And what about red meat? We haven’t been able to afford red meat for quite some time. Why should I deceive? It’s been five or six months since we’ve had any (The words are punctuated with tears)” - (Official Interview with the mother of a working child)*.

*“The situation is not very good for these children. One of our concerns is their health. Now we know how much malnutrition there is and how anemic these children are. Their growth is retarded and their cognitive development is insufficient. Our stories are very deep and complex”* (Official Interview with a social deputy).

#### Sleep disorder and fatigue

In addition to malnutrition, sleep disorders and fatigue emerge as pervasive health concerns affecting working children. Our observations reveal a notable pattern of insufficient quantity and poor quality of sleep, leading to chronic fatigue and physical weakness. These issues are often attributed to the demanding nature of their work, characterized by heavy workloads and long hours, compounded by adverse conditions in the work environment. Such circumstances disrupt children’s sleep patterns and contribute to a general sense of fatigue and debilitation, underscoring the detrimental impact of their labor conditions on their overall health and well-being.

*“The patient was a 7-year-old girl who lives on the outskirts of the city. She was admitted to the hospital because of a dog bite. According to the psychiatrist, after this accident, she had disorders such as anxiety, fear, nightmares and sleep disorders” (*Examining the condition of a working child admitted to the hospital).

*“ P(boy), unfolded the narrative of his restless sleep amid the backdrop of his labor, recounting tales of slumber on pavements and rattling buses. He said that when I get home, everyone is asleep. He said that I am afraid because the electricity is off. He said that although sometimes I get very tired at work and I want to go to sleep earlier, but I can’t fall asleep in bed and I go to sleep very late”* (Informal interactions - street).

#### Inadequate hygiene, impaired health

Inadequate hygiene practices represent a significant health challenge for working children, particularly those hailing from economically and culturally disadvantaged backgrounds. Our research indicates that the combination of low health literacy among families, economic hardships, and prevailing cultural norms engenders numerous hygiene-related issues among these children. Common ailments observed include head lice infestations, oral and dental problems, and various dermatological conditions. These challenges underscore the intersectionality of socioeconomic factors and cultural contexts in shaping the health outcomes of working children, emphasizing the need for targeted interventions to address hygiene disparities and mitigate associated health risks.

*“When I was sent to welfare accommodation, they shaved my hair. - Do you know why? I had lice, there were little white insects on my head”* (Official interview with a working child- girl/14y).

*“H(boy), who was sitting next to my handkerchief shop, began to talk about two girls, who were talking to boys at the intersection. With a completely slang language he said that, boys sometimes have sex with them, and mentioned some names. I said, “what about you?” He said me too. He was 12 years old, but looked more like a 9-10-year-old boy. He said, we don’t have sex with them anymore because they are dirty. Apparently, one of the boys had developed an allergy or skin disease in the genital area after having sex with the girls, and after that, the boys were afraid of having sex with them*” (Informal interactions - street).

*“Accompanied by a social worker from the association, I embarked on a journey to visit the dwelling of one of the working children. With a gentle knock on the door, we ventured into their world, parting the curtain that shielded their reality. What awaited us inside was a stark departure from the sanctuary one would typically envision as a home. It was more like a courtyard with 5 rooms; or better to say, 5 dwellings. These 5 rooms were in different sections with shared bathroom and a kitchen. The yard was crowded with clotheslines and bird cages, making it difficult to walk through. The bathroom was a cramped space, almost the size of a toilet bowl, with a water tap and a plastic hose lying on the ground. There was also a broken washing basin in front of the toilet door, apparently unused for a long time. The kitchen, with a slight level difference, was almost next to the bathroom. The only thing resembling a kitchen was an old and dirty gas stove. There wasn’t even a sink in the kitchen, and they had to wash their dishes in the yard, where they also washed their hands. There were several bird cages in the kitchen, and their droppings were visible everywhere. Apparently, besides the interest in keeping birds, breeding them could also be considered as a source of income for these families. We entered the house we intended to visit. It was right in front of the entrance door, and they had drawn a curtain in front of it. As we entered, there was a grandmother, mother, daughter, and teenage son who had been a student of association, along with a two-year-old daughter in the room. The mother was 31 years old, pregnant, and due soon. When this child is born, their family will grow to 8 members, excluding the grandmother. She said that despite the expenses of a cesarean section, although they have been told that she should have a cesarean, she has decided to give birth in the delivery room; a room that might not be more than six meters. The floor of the room was covered with a six-meter carpet, and there was hardly any empty space around it. There was a refrigerator, heater, and a few pillows as a backrest in the room. Two curtains were also on the walls of the room, one behind the bed and the other behind the television. Her son had pushed aside the curtain and was watching TV; I realized that was probably the only possible place to put the television. The room lay ensconced in an eternal shroud of melancholy, its very essence suffused with the weight of despair. Each corner bore the indelible marks of moisture, like silent tears etched upon the walls. The pallor of dampness had tainted its once-vibrant hues, rendering them a sickly yellow, as if drained of life itself. With every breath, you could almost taste the musty decay that clung to the air, a palpable reminder of the room’s fragility. And yet, despite its evident decrepitude, it stood as a testament to resilience in the face of adversity. Its cramped confines offered scant solace. How could eight individuals find rest in such cramped quarters, where we struggled even to find a place to sit?”* (Visiting the home of a working child).

### Disquieted mind

Beyond the physical ailments, working children grapple with a myriad of psychological challenges, manifesting in a spectrum of issues ranging from anxiety and depression to feelings of isolation and diminished self-esteem. These interconnected struggles collectively constitute a distinct sub-category termed ‘disquieted mind’ in our study.

#### Anxious children

Anxiety emerges as a prevalent psychological disorder affecting working children, with a multitude of stressors contributing to its onset. Factors such as separation from home and family, tardiness at work resulting in reprimand from employers, street harassment, and familial pressures to perform contribute significantly to the anxiety experienced by these children.

*“The family asks him to come and work instead of playing game or watching TV. They put a lot of pressure on him by saying that, come on, do your work, we have to deliver our work on time. This makes the children stressed and forced to work despite their inner desire”* (Official interview with a health official).

*“For example, these children are very stressed because of the conditions they live in. They have to lift heavy loads. I know a child who had heart attack because of these conditions. He had gone to doctor several times and the reason was not clear at all, because he was working, he was moving loads. So, because of that stressful situation, he kept having heart attacks. He even lost consciousness once. Then, they took him to the doctor and after assessment the doctor said there is nothing wrong with him. It was all from stress”* (Official interview with a social worker).

*“Sometimes my brother beats me up. For example, he says you didn’t work well. I always say, God… can I die to get rid of this life?”* (Official interview with a working child- 14y- boy).

*“Z (girl) came and sat next to me and kept turning to herself like someone who has a severe heartache. Said that he did not find his younger sister who was working on the street. She said what should I answer my mother? What if someone stole her? I will be miserable, miserable…”* (Informal interactions - street).

#### Reduced self-esteem

Reduced self-esteem is a prevalent issue among working children, despite their ability to form strong social bonds. Their confidence tends to falter across various contexts, evident in feelings of inadequacy and unworthiness. Often, they harbor a sense of dirtiness or label themselves as beggars, experiencing shame when working on the streets in the company of acquaintances. Additionally, they struggle to perceive themselves as deserving of praise, feeling inherently weak and inferior. Frequent instances of insult and humiliation further erode their self-esteem.

*“As we sat on the cold pavement, lost in conversation, a 29-30-year-old man, his eyes betraying a weariness far beyond his years, appeared before us. With a violin cradled in his arms, he began to sing, his voice carrying the weight of a thousand sorrows (to receive money). At that time, Z (girl) laughed loudly. “Look at us,” she exclaimed, her voice laced with bitter irony. “Here we sit, mere beggars ourselves.”* (Observations- street- 10 PM).

*“One of the problems of these children is their low self-confidence. Reasons such as inappropriate working and living conditions, problems in the work environment, cold, hunger, and being humiliated are among factors that can decrease these children’s self-confidence”* (Official interview with a volunteer physician).

#### Unfocused mind

An unfocused mind represents a significant challenge observed among working children, manifesting in difficulties with concentration and learning. These children frequently exhibit signs of fatigue and intellectual disarray, which hinder their ability to engage effectively in educational pursuits. According to feedback from school teachers and officials, the academic performance of many working children falls below that of their peers, indicating a disparity in learning levels. These observations underscore the detrimental impact of labor-related fatigue and cognitive strain on the educational attainment and cognitive development of working children.

*“I have seen many times in the classroom that the teacher talks to them about different contexts. But they do not pay attention to what the teacher says because they either have been working till late last night or have been beaten, etc. I mean, these children are not mentally and emotionally comfortable to sit in the classroom and learn, so they are weak in the field of learning”* (Official interview with a social worker).

*“They are weak in different fields, because they don’t have physical and mental support, they don’t know social issues well, and they have family problems that follow them even to school. We want to teach them something, but they are not focused as if their body is here, but their mind is somewhere else”* (Official interview with a social worker).

### Disrupted sociability

Disrupted sociability emerges as a prominent feature of the behavioral profile observed among working children in this study. These children exhibit a latent sense of resentment towards the broader society, perceiving themselves as separate from and perhaps marginalized by societal norms. This sentiment manifests in various behaviors indicative of social dysfunction, including bullying, acts of aggression and violence, and engagement in reprehensible social conduct. Additionally, working children demonstrate a tendency to disregard the property rights of others, cultivate strained relationships, and exhibit stubborn and vindictive tendencies. Moreover, they engage in out-group self-censorship, inhibiting their authentic expression within social contexts.

#### Negative social role modeling

Interaction with working children reveals a concerning trend wherein many gravitate towards individuals who exhibit antisocial behaviors as role models. This phenomenon, undoubtedly influenced by the challenging work and living conditions they endure, underscores the complex interplay between environment and behavior. Through close observation of these children and their tendency to repeat similar antisocial behaviors, we found that they consider these norm-breaking behaviors powerfully and interesting.

*“We had a child who said, police were following S (a drug dealer man) and I was watching from the window. The police car came but could not catch S… Do you know what I mean? Children say, police came even with gun, but they could not catch S, because he is a powerful person. This is interesting to them. It is like they want him to be their role model, so that in the future they can become powerful like him. All those police came but could not catch S…”* (Official interview with a social worker).

*“This student used to go out with very bad friends, with people who were doing pickpockets and such. He was going towards negative direction, and I was thinking no matter what I try, I cannot do anything”* (Official interview with a school principal).

#### Aggression and violence

Aggression and violence can be viewed as the one of most obvious characteristics of working children, which is evident in almost all interactions. Many of these children tend to vent their anger in unusual ways. They feel good when they beat others and are used to cursing and rude words. They are often involved in verbal and physical fights among each other, and are aggressive in their games and fights. To convince other children, they resort to force and beating. They often carry with them a latent anger from society’s abuse, and some even carry knives, emblematic of their readiness for potential confrontations.

*“P (boy) took out a knife from his pocket and showed it to me. He said is it beautiful? he queried, a glimmer of pride flickering in his eyes. “Why do you carry such a thing?” I inquired; my voice heavy with concern. “At times, it becomes necessary,” he replied cryptically, his words shrouded in ambiguity. He proceeded to recount a tale of conflict and strife, where he found himself pitted against a menacing gang. In the heat of battle, he wielded a broken bottle with a ferocity born of desperation, his actions a stark testament to the harsh realities of life on the streets. This was not the first time for me seeing knife in the hands of these children, as I had seen knives in F(boy) and H’s(boy) pockets before”* (Informal interactions - street).

*“H (boy) said, people from your city are very cowards, they can’t do anything when they are alone, but when they are together they look for trouble. P (boy) continued, I like to beat them up. I asked him why? He didn’t answer; he said he just likes to beat them up. H (boy) said that, we like to beat everyone, we feel very good when we beat others”* (Informal interactions - street).

*“He (13-year-old boy), in a moment brimming with righteous indignation, voiced his fervent desire for retribution, saying: ‘I would like to go and beat them, those who did not allow the group of children to work in the street, as much as I can. I am not worried about myself; I am afraid that they will also hit M, my brother. He is 5 years old and cannot defend himself”* (Informal interactions - street).

#### Stubbornness and vindictiveness

Stubbornness and vindictiveness are among other behavioral characteristics of many working children. Low-level codes extracted in this study, including a hitter gets hit, revenge of brother from sister in the patriarchal atmosphere of home, damaging and dirtying the car of a driver who hasn’t paid for window cleaning, cursing a cursing driver, defiance and not being amenable can confirm this finding.

*“- One bad thing about people is that, you clean their car’s window, but they do not pay and say to themselves let her clean it. Then when you tell them to pay, they lower the car’s window and say we didn’t ask you to clean it. Go away. Or sometimes we clean the window, and they see but they say they don’t have money to pay. They let the red light turns green without touching their pocket, then they say I don’t have money and leave. - What do you do in that situation? - We make their window dirty again” (*Official interview with a working child- 12y- girl).

#### Harassment

It appears that numerous working children engage in harassing behaviors towards others through diverse means Playing with other people’s feelings and making fun of them, ignoring the presence of people and fighting in the street, playing football on the sidewalk despite the warnings of pedestrians, talking loudly in the subway, abusing animal, and harassing others are among behaviors that working children often display.

*“A(girl) told a story when she was talking loudly with Z(girl) and few other girls in the subway a few days ago, when a young lady warned them and asked them to be quite. She said when the train stopped, I slapped her in the face and ran away from the subway”* (Informal interactions - street).

*“-We had a teacher, and we used to harass her so much that she left our school. We changed three teachers in one year. -What were you doing? -We were harassing her and making loud sound to the point that she left (she was talking happily and with a feeling of strength).” (*Official interview with a working child- 15y- boy).

#### Reprehensible social behaviors

Through extensive, long-term interactions within the context of working children, we have come to recognize a troubling prevalence of reprehensible social behaviors within this group. These behaviors encompass a range of actions, including disregard for laws and authority, actively promoting rioting and vandalism among peers, exhibiting a lack of regard for social norms by urinating in public spaces, and demonstrating a general indifference towards social etiquette and respect. Moreover, there appears to be a pervasive sense of disconnection from civic responsibility, with many working children showing little concern for the health and safety of the city.

*“One of the children came over to me with a sandwich. He put half of it in a plastic bag and gave it to me. I didn’t eat it, and I said I have a cold and if I take it I will get worse. They ate the sandwich together and as usual left the garbage on the street. They throw all the garbage on the ground. I said, is this a trash can? P(boy) said, “our city is our home, the municipality is our servant!” and they laughed together; a laughter in stark contrast to the bitter reality of their surroundings.”* (Observations- street).

“I (a child laborer) am an outlaw, why? I am free, I do whatever I want at the crossroads, I beg at the crossroads, I take money when I want, maybe I even steal at the crossroads” (Official interview with a social worker).

#### Ignoring other’s ownership

Another concerning aspect of socialization among working children is the tendency to disregard others’ ownership rights, evidenced by behaviors such as pickpocketing, theft, and unauthorized use of property. Instances of these behaviors are occasionally observed among working children, reflecting a lack of respect for the property rights of others.

*“H(boy) told the story of a day when he and P(boy) took a bouquet of flowers from a flower shop without permission and sold it. I jokingly asked them, how could you spend such money?! They said God has created money for everyone. I said the bank prints the money not God. He said, well, God has created those devices. I said people have made them and explained to them, it’s like me saying that God created your shoes, so because God created them, they are mine. He laughed and said that God created me with shoes on. H(boy) said God has divided the money among people and we take our share of money from people”* (Informal interactions - street).

#### Disturbed relationships

Working children often experience relationships that diverge significantly from those of the general public, often exhibiting signs of disorder. Such disruptions can be observed within family dynamics, friendships, and community interactions. Examples include intra and extra-group conflicts, disrupted family relationships, and the unfortunate scenario of siblings being viewed as rivals for work opportunities within the household. Additionally, gender discrimination can exacerbate tensions between siblings, further complicating familial dynamics.

*“According to the psychologist, every child here has a file whose source of information is the child himself, parents and teachers. He considered the most important issue of working children to be their communication problem. According to him, working children are weak in communicating with each other and others, and they cannot express their wishes calmly like other children”* (Informal interactions – school).

#### Out-group self-censorship

Working children often perceive themselves as a distinct group from mainstream society, leading to a pervasive sense of mistrust towards individuals outside their immediate circles. Consequently, many of these children exhibit self-censorship when interacting with individuals beyond their group, often presenting a curated version of themselves that may diverge from their authentic behavior. Instances of such out-group self-censorship are evident in various aspects of their conduct, including concealing behaviors such as smoking from school authorities and peers, refraining from using profanity in educational settings, and feigning adherence to hygiene standards at school. Additionally, the fear of repercussions from family and school may prompt working children to remove visible markers such as tattoos after work.

*“One of my colleagues was doing a research on physical child abuse, so we listed a bunch of children for her to study. When she went to talk to the children, they were telling that their fathers always buy them the best things, their mothers buy them the best dolls and make the best foods for them, and they have the best relationships, etc. My colleague asked me why is it like that? I said, look, as we enter children’s homes they can’t hide anything from us, but if a stranger talk to them, they won’t give correct information”* (Official interview with a social worker).

*“M(boy) said to me; aunty, this (a boy about six or seven years old) have broken the record today. He looked at the boy and said; tell how much did you sell today?! They boy looked at me and didn’t respond. A told him that aunty is one of us and she is not a stranger. Then, the boy talked about how much handkerchiefs he had sold that day”* (Informal interactions - Street).

## Discussion

The findings of this study highlight three categories of tormented body, disquieted mind and disrupted sociability as the main factors that contribute to the impaired health of working children. Carsepkan recommends that in the last stage of research, the findings should be explained in the form of macro theories, and micro findings should be analyzed through sociological findings [11]. To execute this step in the present research, we scrutinized some prior studies and existing theories closely related with the research findings.

Based on the tormented body sub-category, this study found that many working children experience various physical injuries. Ahmadi (2021) reported that working children often engage in fights and endure long walks to work, exposing them to extreme weather conditions and pollutants. They also suffer from accidents, falls, and cuts during play, aligning with our study’s findings [12]. Navile (2014) observed that working children in Bangladesh face hand and foot injuries, accidents, respiratory and digestive disorders, headaches, skin ailments, hepatitis, back pain, and falls, mirroring our results [13].

Nerg (2016) found that Zambian working children face traffic accidents, communicable diseases, substance addiction, respiratory infections due to harsh weather, involvement in prostitution, lack of sexual education, heavy lifting, and limited healthcare access. They also suffer from exploitation and harassment [14]. Similarly, Fahmy Mohamed et al. reported that 90% of children in industrial workshops face issues like body pain, skin problems, loss of appetite, and ear, eye, and throat ailments [15]. Jalili Moayad et al. (2021) documented that 77.6% of working children in Iran experience emotional, physical, or sexual abuse [7].

Posso categorized child labor into hazardous and non-hazardous activities, finding that children in hazardous jobs face more health issues like respiratory problems, body aches, and wounds, compared to those in non-hazardous jobs and non-working children. However, Posso suggested that child labor generally leads to adverse health outcomes [16]. Our study supports this, indicating that all working children face health problems, regardless of their work type.

Based on the sub-category of a disquieted mind, working children experience many psychological problems such as anxiety, depression, and isolation, reduced self-esteem, and an unfocused mind. Nerg showed that working children have many psychological problems, including a lack of support, a sense of insecurity due to violence, and stigma [14]. These findings align with the outcomes of the current study. Hamdan-Mansour (2013) in a study in Jordan argued that working is associated with missing school, loss of learning and playing time, and interaction with peers. In addition, working in a job that is considered humiliating or inferior can lead to peer rejection and lower self-esteem [17]. In another study, Ahmed and colleagues compared the physical health, psychosocial health, and quality of life of 45 working children in their study and showed that working children had lower mean scores of physical and psychosocial health than children in the control group. They also showed a significant difference in the quality of life of children in the intervention and control groups [18]. Although such studies conducted with a quantitative approach cannot be accurately compared with qualitative studies, our study has also shown problems related to the physical, mental, and social health of working children.

Based on the disrupted sociability sub-category, working children harbor latent anger towards the general public and perceive themselves as distinct from it. Manuel Castells, in his study “The Information Age,” examines the restructuring of global capitalism and the rise of informational capitalism, highlighting negative impacts such as social exclusion and marginalization resulting from power relations and investment. Social exclusion, characterized by limited access to social rights and discrimination based on various factors, impedes individuals and communities from fully participating in societal activities. This leads to intensified exploitation and marginalization, particularly evident in the exploitation of children. Castells’ research underscores how global economic disparities exacerbate these issues, resulting in poverty, inequality, and the emergence of an informal economy, which disproportionately affects women and children [12, 19]. Balali’s study reinforces these findings, emphasizing the societal costs of exclusion and the potential for individuals to resort to crime or violence as a response [20]. This aligns with observations of working children experiencing stigma and engaging in destructive behaviors due to societal discrimination. In another study, Asante (2016) refers to factors such as violence, violent behavior, harassment and sexual assault, alcohol and drug use, and social stigma in relation to working children [21]. These items align closely with the current study and substantiate its findings.

### Limitations of the study

In ethnographic studies, since the researcher spends time observing the interaction, he or she may be considered an outsider that may cause a change in the behaviors of samples under the study. Although Carsepkan considers this issue to a part of culture that governs group activity [11], in order to overcome this problem in this study, the researcher tried to participate in the activities of working children as a group member while observing their behavior. In addition, in order to gain trust, in the first weeks of the study, the researcher only played the role of “observer” or “participant observer” and conducted no interview with the working children. Also, despite the effort to comprehensively examine the lives of working children, coexistence with working children was not possible after 12 o’clock midnight due to the risks that this could have for the researcher. So, these lost times were investigated by conducting interviews with the children and using other research methods.

## Conclusions

The results of this study showed that the health of working children is impaired in different physical, psychological and social ways. Since the main results of this critical ethnographic study show the disturbed health of working children, changes are necessary to improve the health of these children. It seems that the most important measure that can be taken in this regard is to set a universal legal status, according to which all children, regardless of gender, age, race, ethnicity, religion and citizenship have all the rights and free access to educational and health facilities. Also, due to the fact that one of the causes of child labor is the lack of knowledge about their rights, it is necessary to help children (working and non-working children) to learn about their rights under the law, using textbooks, educational courses and workshops, children’s programs, etc.

## Data Availability

The datasets used and analyzed during the current study available from the corresponding author on reasonable request.

## Abbreviations

TOMAN: A currency in Iran
TUMS: Tehran University of Medical Sciences
